# Prediction of presence and severity of metabolic syndrome using regional body volumes measured by a multisensor white-light 3D scanner and validation using a mobile technology

**DOI:** 10.1093/ehjdh/ztae059

**Published:** 2024-08-15

**Authors:** Betsy J Medina Inojosa, Virend K Somers, Kyla Lara-Breitinger, Lynne A Johnson, Jose R Medina-Inojosa, Francisco Lopez-Jimenez

**Affiliations:** Division of Preventive Cardiology, Department of Cardiovascular Medicine, Mayo Clinic, 200 First Street SW, Rochester, MN 55905, USA; Division of Preventive Cardiology, Department of Cardiovascular Medicine, Mayo Clinic, 200 First Street SW, Rochester, MN 55905, USA; Division of Preventive Cardiology, Department of Cardiovascular Medicine, Mayo Clinic, 200 First Street SW, Rochester, MN 55905, USA; Dan Abraham Healthy Living Center, Mayo Clinic, 200 First Street SW, Rochester, MN 55905, USA; Dan Abraham Healthy Living Center, Mayo Clinic, 200 First Street SW, Rochester, MN 55905, USA; Division of Preventive Cardiology, Department of Cardiovascular Medicine, Mayo Clinic, 200 First Street SW, Rochester, MN 55905, USA; Division of Epidemiology, Department of Quantitative Health Sciences, Mayo Clinic, 200 First Street SW, Rochester, MN 55905, USA; Division of Preventive Cardiology, Department of Cardiovascular Medicine, Mayo Clinic, 200 First Street SW, Rochester, MN 55905, USA; Dan Abraham Healthy Living Center, Mayo Clinic, 200 First Street SW, Rochester, MN 55905, USA

**Keywords:** Metabolic syndrome, Body fat, Body mass index, Body volumes, 3D scan, Obesity

## Abstract

**Aims:**

To test whether an index based on the combination of demographics and body volumes obtained with a multisensor 3D body volume (3D-BV) scanner and biplane imaging using a mobile application (myBVI®) will reliably predict the severity and presence of metabolic syndrome (MS).

**Methods and results:**

We enrolled 1280 consecutive subjects who completed study protocol measurements, including 3D-BV and myBVI®. Body volumes and demographics were screened using the least absolute shrinkage and selection operator to select features associated with an MS severity score and prevalence. We randomly selected 80% of the subjects to train the models, and performance was assessed in 20% of the remaining observations and externally validated on 133 volunteers who prospectively underwent myBVI® measurements. The mean ± SD age was 43.7 ± 12.2 years, 63.7% were women, body mass index (BMI) was 28.2 ± 6.2 kg/m^2^, and 30.2% had MS and an MS severity *z*-score of −0.2 ± 0.9. Features *β* coefficients equal to zero were removed from the model, and 14 were included in the final model and used to calculate the body volume index (BVI), demonstrating an area under the receiving operating curve (AUC) of 0.83 in the validation set. The myBVI® cohort had a mean age of 33 ± 10.3 years, 61% of whom were women, 10.5% MS, an average MS severity *z*-score of −0.8, and an AUC of 0.88.

**Conclusion:**

The described BVI model was associated with an increased severity and prevalence of MS compared with BMI and waist-to-hip ratio. Validation of the BVI had excellent performance when using myBVI®. This model could serve as a powerful screening tool for identifying MS.

## Graphical Abstract

**Graphical Abstract:**
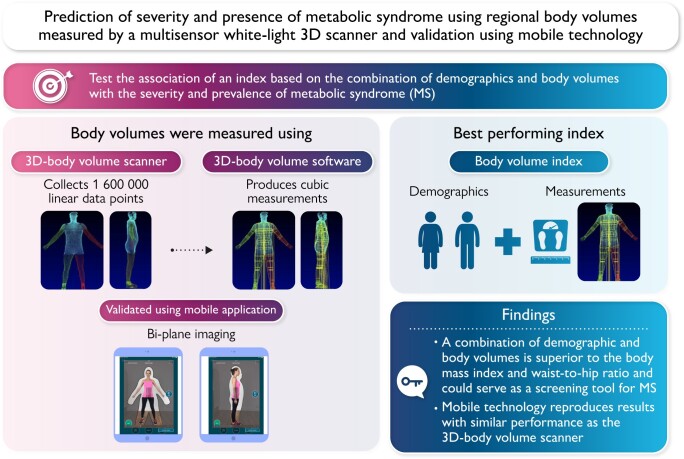

## Introduction

Metabolic syndrome (MS) is a constellation of adiposity-related cardiometabolic risk factors (e.g. central obesity, dyslipidaemia, hyperglycaemia, and hypertension) strongly associated with Type 2 diabetes incidence, poor cardiovascular disease (CVD) outcomes, and non-CVD complications (i.e. various forms of cancer, liver disease, cirrhosis, pancreatitis, cognitive decline, and dementia).^1,2^ Recognition and accurate MS risk stratification are essential to guide early preventative interventions.^2^ The global burden of metabolic syndrome is on the rise, almost reaching hyperendemic status, with an estimated prevalence rate of 41.8% in the USA, disproportionately affecting those with lower educational levels, with low socioeconomic status, and who experienced food insecurity.^1,3^

Due to strong epidemiological and clinical validity, the most accepted criteria to characterize MS are proposed by the National Cholesterol Education Program Adult Treatment Panel III (NCEP-ATPIII),^4^ and the panel defines MS as the presence of three or more of the following criteria [large waist circumference (WC), high triglyceride level, reduced HDL cholesterol, elevated fasting glucose, and increased blood pressure].^2,5^ Nevertheless, there is growing recognition of MS as a disease spectrum, and if the differences in clinical profiles between sex and racial/ethnic groups are added to this aspect, it leads to the derivation of the MS severity *z*-score, which correlates with the future risk of CVD and Type 2 diabetes mellitus.^4,6^

Clinically, tape-measured metrics such as the WC and waist-to-hip ratio (WHR) outline central obesity as these are widely used surrogate markers of abdominal adiposity and fat distribution that represent key determinants of worse cardiometabolic risk profiles and mortality, and are recommended to be used along with body mass index (BMI).^7,8^ These measurements have limited acceptance in clinical practice and research as they may lead to the misclassification of central obesity, given reports of inaccuracy and intra- and interobserver variability requiring trained personnel and consequently affecting key MS criteria.^9^

A multisensor 3D body volume (3D-BV) scanner (Select Research; Worcester, UK), a non-invasive device used in the clothing industry to assess body shape and size, is a reliable and valid technique for determining body circumferences and composition.^10,11^ Moreover, our group recently validated a mobile application (myBVI®, Select Research) that leverages biplane imaging (i.e. front- and side-facing photographs) ascertained with a mobile technology to accurately determine body volumes (BVs),^12^ the use of camera-enabled technology in the medical field is increasingly common, with growing validation and implementation,^13–15^ representing a promising portable, affordable, and scalable tool with wide applicability in clinical settings, including telemedicine.

In this study, our primary aim is to assess the association between an index or a combination of demographics and BV obtained with a 3D-BV scanner and the severity and prevalence of MS among attendees of an employee wellness centre. Our secondary aim is to validate the resulting MS prediction model using volumes obtained from biplane imaging with a mobile application (myBVI®).

## Methods

### Study population

The Mayo Clinic Institutional Review Board (IRB) approved the study protocol as a minimal risk study. Over an 8-month period, consecutive volunteers over 18 years of age underwent a physical fitness examination as part of their attendance at an employee wellness centre. Evaluations consisted of standardized clinical questionnaires, blood tests, and anthropometric measurements, including 3D-BV ascertainment. For our secondary aim, after obtaining written informed consent, we prospectively recruited volunteers to participate in the study using IRB-approved materials, to undergo 3D-BV and biplane imaging employing a mobile application (explained in the Mobile device measurements section). According to the study protocol, we excluded patients with claustrophobia, those who were unable to stand still during measurements, and those who did not provide research authorization.

### Anthropometric measurements

All subjects underwent a protocolized anthropometric evaluation, including height recorded by standing barefoot to the nearest centimetre using a stadiometer (Seca; Hanover, MD, USA) and body weight recorded to the nearest 0.1 kg with a high sensitivity scale (Tanita Corporation; Arlington Heights, IL, USA). Participants underwent study measurements wearing form-fitted gym clothing or undergarments to accurately capture body shape and size; long hair was raised to expose the neck.

### 3D scanning measurements

**Figure 1:**
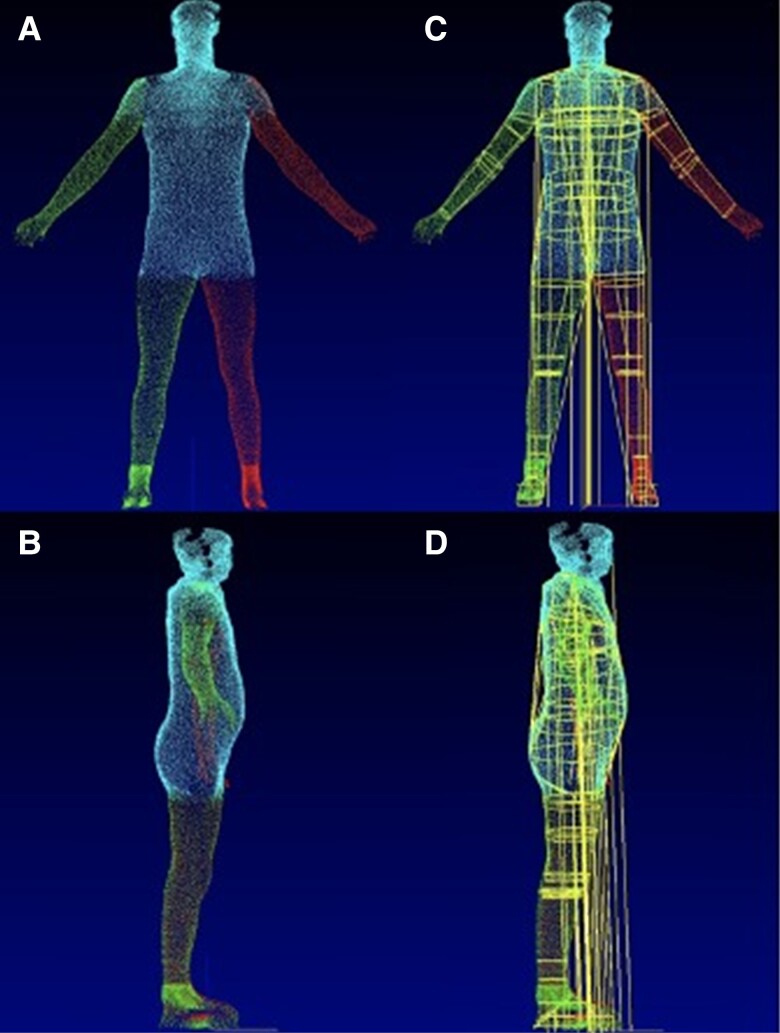
(*A*) Front-facing and (*B*) side-facing images taken by the multisensor white-light 3D body volume scanner (Select Research). (*C* and *D*) Post-processing of images used to estimate and derive body volumes.

Body volume measurements were obtained using a multisensor white-light 3D-BV scanner version 2.1 (*Figure 1*) (Select Research). The protocol for 3D-BV scanning has been previously described in detail.^10–12^ In brief, subjects were instructed to stand facing forward and motionless, with both feet on standard landmarks (centred 60 cm from the front scanner wall). The scanner uses 32 cameras, forming 16 sensors (located at 4 angles at 4 heights) to collect 1 600 000 linear data points over the scanning field. The 3D-BV software utilizes the obtained data points to produce cubic measurements at each cross section with a point accuracy of <1 mm^3^ over the scan field during the 7 s of scanning time. Proper calibration of the 3D-BV scanning system to measure circumferences was performed before each measurement session by using a cylinder with a known volume as the reference standard. Data on regional BV were extracted without alteration from the device’s software.

### Mobile device measurements

**Figure 2:**
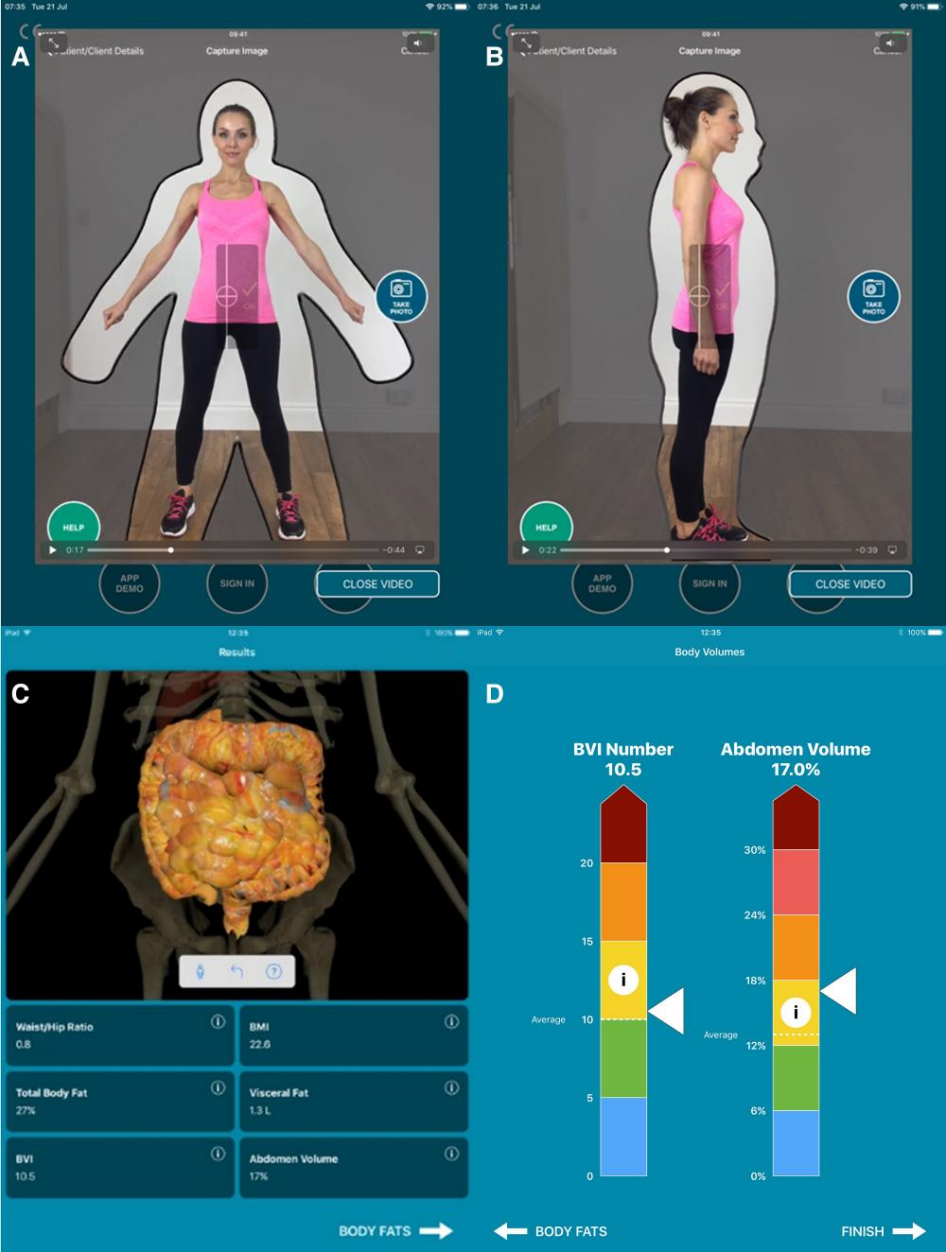
(*A*) Front-facing and (*B*) side-facing images taken by the myBVI® mobile application. (*C* and *D*) myBVI® application results.

For the mobile device measurements, 133 volunteers were asked to stand in an anatomical pose at precisely 2 m from the mobile device. Photographs were obtained with an iPad® through a proprietary application [myBVI® (Body Composition Calculator) application, available at the Apple App Store, version 2.4.0; Select Research]. To ensure consistency, the iPad® accelerometer was used to guarantee that the device was held upright with no tilt. A viewfinder overlay was used to ensure the correct anatomical pose with arms and legs at the required angles and correct subject positioning. (*Figure 2*) For example, if the subject was small in the frame, the operator moved the device closer to the subject. Front-facing and side-facing images were taken in a standardized testing environment (examination room) standing against a plain background with light-emitting diode lighting, guaranteeing even illuminated images with minimal glare, shadows, and adequate contrast. All measurements were taken by trained investigators who were masked to the final application output. Further details have been published previously.^12^

### Metabolic syndrome

Metabolic syndrome prevalence was defined as ≥3 of 5 metrics according to the NCEP-ATPIII criteria (*Table 1*).^2^ Disease severity was determined by calculating the MS severity *z*-score using sex and racial/ethnic-based models as described previously.^6,16^ Baseline characteristics of the study subjects were extracted from the medical record in relation to the evaluation date.

**Table 1:** Definition of metabolic syndrome according to the National Center for Environmental Health and Safety (NCEP) ATP III criteria

Metric	Definition
Large waist	>35 inches (>88 cm) for women and >40 inches (>102 cm) for men
High triglyceride level	≥150 mg/dL (≥1.7 mmol/L)
Reduced HDL cholesterol	<40 mg/dL (<1.04 mmol/L) in men or <50 mg/dL (<1.3 mmol/L) in women
Increased blood pressure	≥130/85 mmHg
Elevated fasting glucose	≥100 mg/dL (≥5.6 mmol/L)

### Statistical analysis

Subject characteristics are described as frequencies with percentages, mean values, and standard deviation, without formal statistical comparison per study design. Aiming to select features associated with MS severity,^6^ BV and demographic variables were screened using the least absolute shrinkage and selection operator (LASSO) regression.^17,18^ Variables with estimates equal to zero were screened out of the final model. The body volume index (BVI) model was developed using a random sample of 80% of subjects (training set). Then, the resulting predicted probability in continuous form was modelled using a threshold that balanced sensitivity and specificity to predict MS. Performance was assessed in (i) 20% of the remaining observations (validation set) and (ii) volunteers who prospectively underwent mobile device (myBVI®) measurements, which served as further independent external validation. An additional exploratory analysis aimed to assess model performance across levels of disease burden reported performance while predicting the number of MS components (e.g. 2, 3, 4, and 5). Performance metrics include area under the receiving operating curve (AUC), sensitivity, specificity, positive predictive value, negative predictive value (NPV), accuracy, and the Akaike information criterion corrected (AICc). We tested the correlation between the BVI model score and the MS components using Pearson’s correlation coefficient (*r*^2^); the results were summarized and illustrated using scatter plots. The level of statistical significance was set at 0.05 for all tests. All statistical analyses were performed using JMP® version 17 (SAS Institute Inc., Cary, NC, USA).

## Results

A total of 1280 subjects were included in the model development, of whom 1024 subjects (80%) were included in the training set and 256 (20%) in the validation set. The overall mean ± SD age was 43.7 ± 12.2 years; over half of the subjects were women (63.7%) and predominantly non-Hispanic white (96%). In the anthropometric measurements, the average BMI was 28.2 ± 6.2 kg/m^2^, WC was 95.7 ± 16.7 cm, WHR was 0.9 ± 0.1, and MS was present in 387 (30.2%), according to the NCEP-ATPIII criteria, with 1.8 ± 1.5 MS criteria met, and an MS severity *z*-score based on the waistline of −0.2 ± 0.9 (*Table 2*).

**Table 2:** Subject baseline characteristics of the development and validation subsets

	Training (*n* = 1024)	Validation (*n* = 256)	Total (*n* = 1280)	Mobile application validation (*n* = 133)
**Demographics**				
Age, years	43.5 ± 12.3	44.6 ± 12.0	43.7 ± 12.2	33 ± 12.8
**Sex**				
Female	646 (63.1%)	169 (66.0%)	815 (63.7%)	77 (58%)
Male	378 (36.9%)	87 (34.0%)	465 (36.3%)	56 (42%)
**Race**				
Non-Hispanic white	984 (96.1%)	244 (95.3%)	1228 (95.9%)	98 (74.0%)
Non-Hispanic black	17 (1.7%)	5 (2.0%)	22 (1.7%)	19 (14.6%)
Hispanic	23 (2.2%)	7 (2.7%)	30 (2.3%)	16 (11.4)
**Laboratory measurements**				
Glucose, mg/dL	99.7 ± 23.6	98.8 ± 18.0	99.5 ± 22.6	93.0 ± 14.8
HDL, mg/dL	59.2 ± 18.0	59.0 ± 17.9	59.2 ± 18.0	61.9 ± 16.6
Triglyceride, mg/dL	114.5 ± 75.2	115.2 ± 67.3	114.6 ± 73.7	85.9 ± 47.5
**Anthropometric measurements**				
Weight, kg	81.9 ± 20.7	80.6 ± 19.7	81.6 ± 20.5	70.7 ± 14.7
Height, m	1.7 ± 0.1	1.7 ± 0.1	1.70 ± 0.1	170.1 ± 9.8
Body mass index, kg/m^2^	28.2 ± 6.3	27.9 ± 6.1	28.2 ± 6.2	25.3 ± 4.4
Waist circumference, cm	95.8 ± 16.7	95.4 ± 16.7	95.7 ± 16.7	79.8 ± 16.3
Waist-to-hip ratio	0.9 ± 0.1	0.9 ± 0.1	0.9 ± 0.1	0.8 ± 0.1
**Metabolic syndrome criteria**				
Elevated fasting glucose	378 (36.9%)	101 (39.4%)	479 (37.4%)	24(18.3%)
Reduced HDL cholesterol	387 (37.8%)	97 (37.9%)	484 (37.8%)	14(10.8%)
High triglycerides	360 (35.2%)	100 (39.1%)	460 (35.9%)	18(13.5%)
Large waist	496 (48.4%)	121 (47.27%)	617 (48.2%)	44(33.3%)
Increased blood pressure	171 (16.7%)	46 (18.0%)	217 (17%)	11(8.6%)
Number of MS criteria	1.8 ± 1.5	1.8 ± 1.5	1.8 ± 1.5	1.0 ± 1.1
MS according to the NCEP-ATPIII criteria	307(29.9%)	80(31.2%)	387 (30.2%)	11 (8.3%)
MS *z*-score	−0.2 ± 0.9	−0.2 ± 0.9	−0.2 ± 0.9	−0.8 ± 0.9
MS percentile	44.1 ± 26.8	44.4 ± 26.2	44.1 ± 26.7	25.9 ± 25.3

Values are mean ± SD, frequency, and (%).

HDL, high-density lipoprotein; MS, metabolic syndrome; NCEP-ATPIII, National Cholesterol Education Program Adult Treatment Panel III.

**Figure 3:**
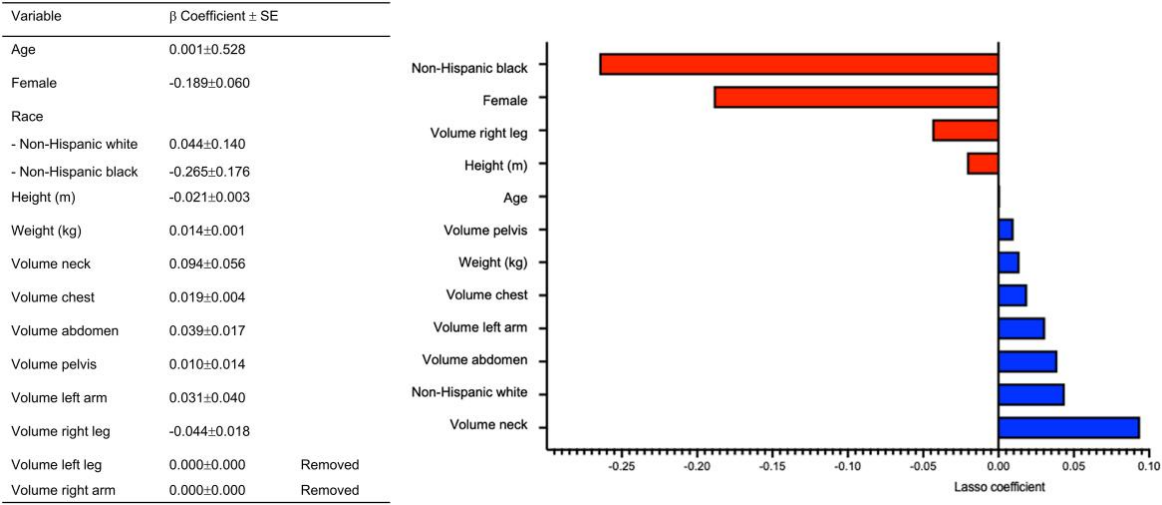
β Coefficients of the features from the least absolute shrinkage and selection operator regressions that are used in the body volume index.

We included 16 features for feature selection by the LASSO, encompassing a combination of eight raw BVs ascertained with the 3D-BV scanning system, height, weight, and demographic characteristics (e.g. age, sex, and race). Features *β* coefficients were obtained at a *λ* value of 0.04410, and 14 were included in the final model. These coefficients were used to calculate the BVI as an addition of the multiplication of each coefficient by each feature value, which is summarized in *Figure 3*.

**Figure 4:**
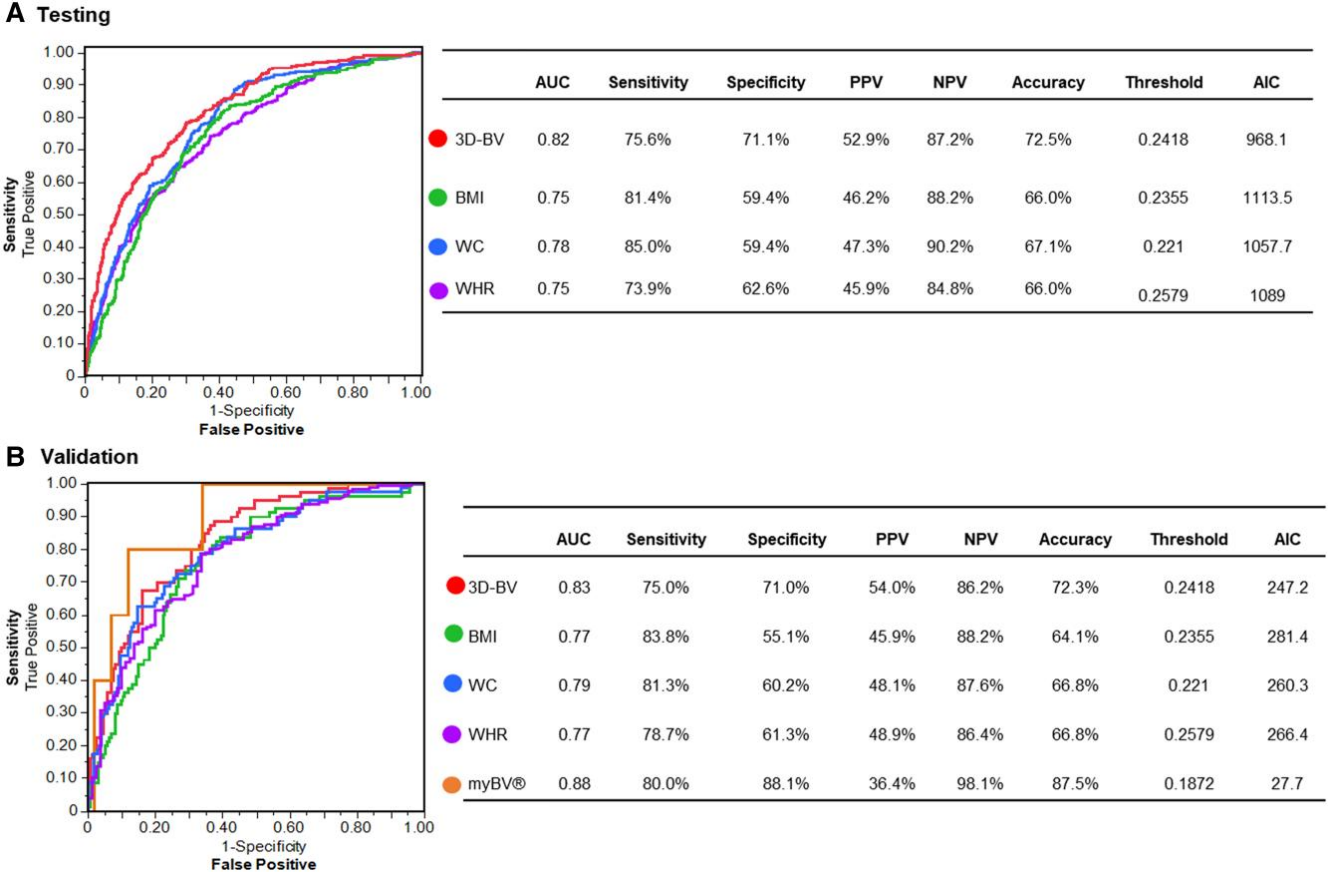
Performance measures of the body volume index model predicting metabolic syndrome by using a 3D body volume scanner and a mobile application compared with conventional anthropometric measurements. Receiver operating characteristic curve and resulting area under the curve of the body volume index for identifying subjects with metabolic syndrome measured with a 3D body volume scanner, a mobile application, and conventional anthropometric measurements. Sensitivity, specificity, and accuracy for detecting metabolic syndrome while using body volume index quantified with a 3D body volume scanner, a mobile application (myBVI®), and conventional anthropometric measurements. (*A*) The testing set included 1024 subjects. (*B*) The validation set included 256 subjects, and external validation of the model was performed using myBVI® on 133 subjects. AIC, akaike information criterion; AUC, area under the receiving operating curve; 3D-BV, 3D body volume scanner; BMI, body mass index; myBVI®, mobile application; NPV, negative predictive value; PPV, positive predictive value; WC, waist circumference; WHR, waist-to-hip ratio.

**Figure 5:**
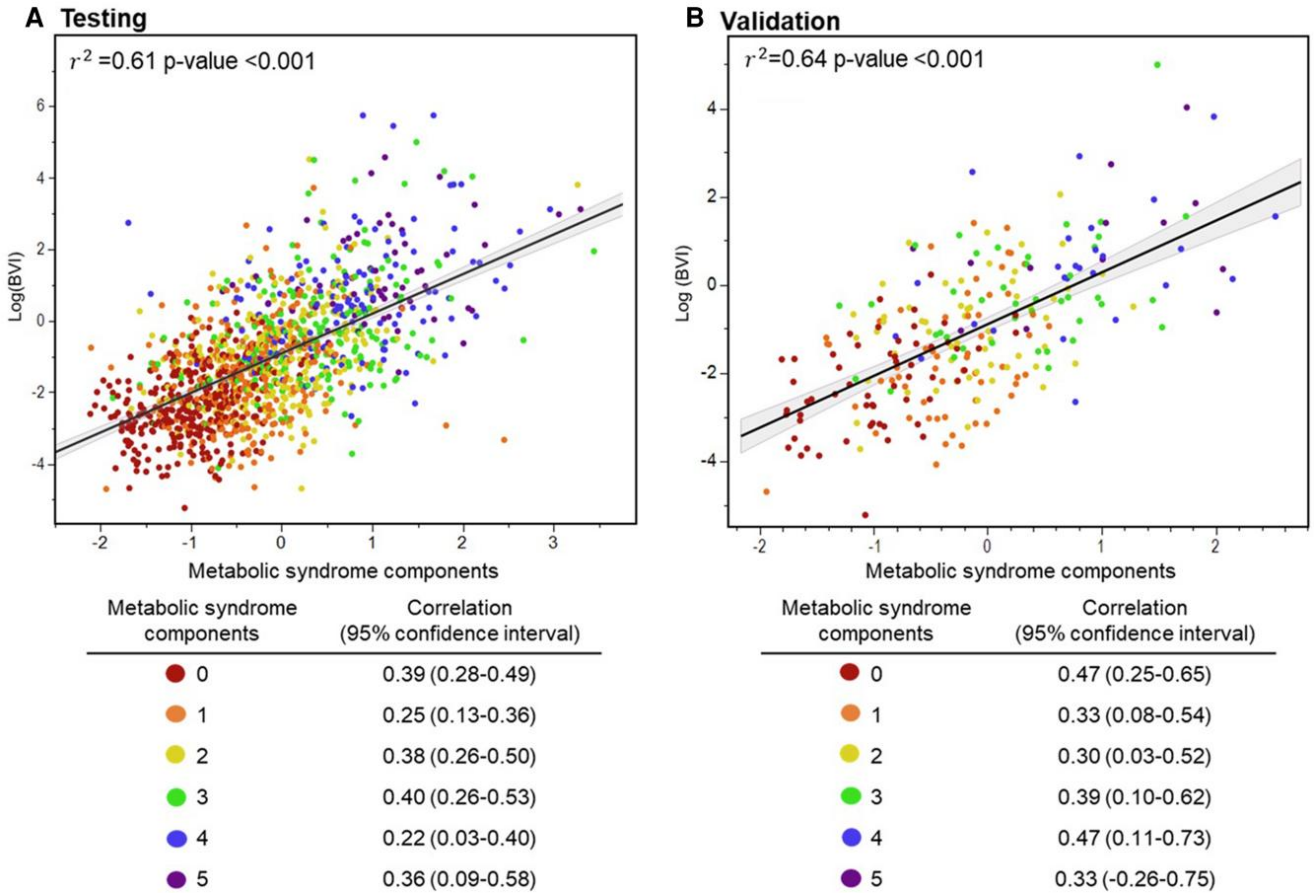
Performance measures of the body volume index model predicting the severity of metabolic syndrome by using a body volume scanner and a mobile application compared with conventional anthropometric measurements. (*A*) The testing set included 1024 subjects. (*B*) The validation set included 256 subjects.

The models’ AUC of the ROC is presented in *Figure 4*. The AUC for the 3D-BV model is 0.83, demonstrating better performance compared with 0.77 for BMI and WHR, in contrast to 0.79 for WC in the validation set (*Figure 4*). The BVI demonstrated a good correlation with the MS components (validation *r*^2^ = 0.64, testing *r*^2^ = 0.61; both *P*-value <0.001) (*Figure 5*). We found improved diagnostic performance sensitivity, NPV, and accuracy with respect to increased MS components; there was no effect on specificity (*Table 3*).

**Table 3:** Performance of the body volume index model predicting metabolic syndrome components

MS components	Sensitivity (%)	Specificity (%)	PPV (%)	NPV (%)	Accuracy (%)
**2**	56.1	76.5	53.3	78.4	69.9
**3**	75.6	76.5	52.5	90.1	76.2
**4**	84.6	76.5	44.0	95.8	77.9
**5**	100	76.5	31.7	100	78.8

MS, metabolic syndrome; NPV, negative predictive value; PPP, positive predictive value;.

### Model validation using mobile devices

External validation was performed on 133 subjects, with the mean age ± SD of 33 ± 12.7 years, of whom 58% were women and predominantly non-Hispanic white race with 98 (74.0%). The average BMI was 25.3 ± 4.4 kg/m^2^, WC was 79.8 ± 16.3 cm, WHR was 0.8 ± 0.1, and MS was present in 8.5%, according to the NCEP-ATPIII criteria, with an average of 1 ± 1.1 met, and an average MS severity *z*-score of −0.8, as shown in *Table 2*. External validation of the BVI prediction model with a mobile technology had an AUC of 0.89 ROC, and diagnostic performance measures are presented in *Figure 3*.

## Discussion

In this study, we demonstrate how volumes obtained by using a non-invasive and mobile 3D-BV technology could serve as a screening tool for MS prevalence and severity. We describe a model that incorporates regional BV and accurately predicts MS prevalence and severity with an excellent prediction performance when compared with conventional anthropometric assessment. Our analyses demonstrate that the BVI model outperformed traditional metrics such as BMI and WHR but also exhibited a lower AICc in comparison with these other prediction paradigms. This suggests that the BVI model achieves a better balance between model complexity and goodness of fit within our sample. Furthermore, external validation of the resulting model using a mobile application demonstrated comparable performance with the potential for wide scalability.

Our findings align with previous analyses performed in various populations that mostly used different non-portable 3D-BV scanning systems (i.e. optical or laser scanners) with essential differences to highlight. Bennett *et al*.^19^ compared a single non-portable 3D optical scanner to BMI and found improvements in MS prediction (AUC for BMI vs. 3D optical scanning of 0.8 and 0.9, respectively), including a complex best-performing (AUC 0.9) final model that required an extensive set of variables (bone mass percentage, left calf volume, WC, and WHR) and was limited by relying on BMI as a significant predictor. Lin *et al*.^20^ also combined regional BV with other anthropometric measurements (i.e. body weight, body height, waist, breast, and hip area). They developed an index that was associated with a higher prevalence of MS. However, unlike the BV, this model had limited portability and required comprehensive anthropometric assessment. Oh *et al*.^21^ used a similar laser-based non-portable laser 3D-BV scanner in a limited sample of Korean women. Correspondingly, they found that each litre increase in BV was associated with MS risk (odds ratio 1.0, 95% confidence interval 1.0–1.2). Chiou *et al*.^22^ combined BMI and WHR components obtained from a non-portable 3D-BV scanner, and although this assessment correlated with components of MS, it did not actually improve diagnostic performance.

The World Healthcare Organization (WHO) has been recognizing the increasing use of mobile technology in health care, especially since the COVID-19 pandemic.^23^ Given the growing availability of mobile devices, the deployment of mobile applications enables new opportunities to facilitate the prevention, monitoring, and management of cardiometabolic conditions such as the MS.^23^ Several applications assess surface dimensions and estimate body circumferences and adiposity with improving precision and accuracy similar to traditional techniques such as BMI, WHR, 3D-BV scanner, and other reference methods.^13–15,24^ However, to the best of our knowledge, this is the first study to develop and validate an MS prediction model using BV ascertained with a mobile application.

Our study has several strengths that warrant consideration. Instead of implementing a traditional binomial classification model for MS prediction, we incorporated the previously validated MS severity *z*-score reported by Gurka *et al*.^6^ mitigating the risk of disease misclassification, potentially enhancing MS screening and informing clinical practice. Our protocolized body composition measurements limited assessment errors and disease misclassification, and our sample size was larger when compared with other studies reporting on similar technology or models.^2,10^ Although non-portable, the 3D-BV scanner is non-invasive and has been previously validated to assess BVs against plethysmography which is considered one of the gold standards in the field. Lastly, the implemented LASSO^17,18^ protocol allows for the optimization of feature selection and data partition, which allows validation of the model in data ascertained with 3D-BV, and we externally validated our prediction model with volumes obtained prospectively using a mobile technology highlighting the potential role of this technology in clinical and research practice. This technology is accessible, cost-effective, and easy to use and can facilitate initial evaluation as well as longitudinal patient follow-up, serving as a potential tool to reduce disparities in access to health care.^13,23^

The diagnosis of MS is centred around laboratory tests, blood pressure, and anthropometric measurements, which are not always feasible due to their unavailability in certain clinical settings, limited accuracy, and low reproducibility.^9^ While there are no routine screening strategies widely accepted for the screening of MS, digital health technologies are promising as they are portable, practical, inexpensive, accessible, and scalable with the potential applicability in remote settings, low-income countries, and large-scale populations while enhancing telemedicine and telecare services.^25^

Among the limitations to recognize include that this study was primarily conducted among white subjects undergoing a wellness evaluation, which reduces the generalizability of our findings. However, the prevalence of MS in our sample is comparable to the latest reports of 34% of the US population.^26^ Additionally, the cross-sectional nature of our study limits the evaluation of possible changes in body composition or distributions and how this might actually affect prognosis.^27^ Also, the relationship between BVI and future CV events was not evaluated, but given the relationship between MS and CVD, the question arises: is it possible that BVI may help predict CV events?^2,28,29^ Finally, although 3D scanning has proved to be an accurate technique to assess body composition and is affordable, the availability of 3D scanners in every clinical environment may be limited compared with traditional anthropometric measurements, restricting the reproducibility of this analysis in other settings.

## Conclusions

Regional BV obtained by using 3D-BV was associated with the severity and prevalence of MS. Validation of the BVI prediction model demonstrated adequate prediction in an external cohort using a mobile technology. These measures may serve as screening tools for MS. However, their utility in clinical practice remains to be evaluated.

## Author contribution

F.L.-J. is a guarantor of this study. J.R.M.-I. and F.L.-J. were responsible for the study conception and design. J.R.M.-I., K.L.-B., and L.A.J. carried out the study. B.J.M.I., V.K.S., J.R.M.-I., L.A.J., K.L.-B., and F.L.-J. interpreted the data. J.R.M.-I. and B.J.M.I. completed statistical analyses and incorporated edits. B.J.M.I., K.L.-B., and J.R.M.-I. were responsible for drafting the manuscript. K.L.-B., B.J.M.I., V.K.S., J.R.M.-I., L.A.J., K.L.-B., and F.L.-J. edited the manuscript. All authors have approved the final version of the manuscript.

## Data Availability

In consideration of the study, subjects’ privacy, study data, and the analytic methods underlying this article cannot be shared publicly. Data can be shared upon a reasonable request to the corresponding author, after approval from Mayo Clinic.
